# D-allose alleviates ischemia/reperfusion (I/R) injury in skin flap via MKP-1

**DOI:** 10.1186/s10020-020-0138-6

**Published:** 2020-02-11

**Authors:** Jihui Ju, Ruixing Hou, Ping Zhang

**Affiliations:** Department of Hand Surgery, Ruihua Affiliated Hospital of Soochow University, No. 5, Tayun Road, Suzhou, 215104 Jiangsu China

**Keywords:** D-allose, Ischemia/reperfusion, ERK1/2, Skin flap, Protection

## Abstract

**Background:**

D-allose was promising in the protection of ischemia/reperfusion (I/R) injury. We intended to investigate the function of D-allose in skin flap of rat followed by the injury of I/R and whether ERK signal pathway was involved in.

**Methods:**

The back flap of Wistar rats was picked up with a vascular bundle of the lateral chest wall. I/R model was made by the venous clamp for 6 h. Rats received D-allose and PD-98059, the inhibitor of ERK1/2, 30 min before modeling. Morphology of tissue was observed by HE staining. Nitric oxide (NO), myeloperoxidase (MPO), malondialdehyde (MDA) and superoxide dismutase (SOD) levels in skin flap were determined by ELISA kits. mRNA and protein levels were determined by qPCR and Western blot respectively.

**Results:**

D-allose alleviated the condition of pathological changes and raised the survival rate of skin flap injured by I/R. Moreover, D-allose suppressed NO, MPO and MDA while elevated SOD levels during I/R status. Furthermore, D-allose decreased MCP-1, TNF-α, IL-1β and IL-6 levels in skin flap injured by I/R. In addition, D-allose inhibited MKP-1 expression and activated ERK1/2 pathway in skin flap injured by I/R. PD-98059 partially counteracted D-allose effects on I/R injury.

**Conclusions:**

D-allose exerted its protective function via inhibiting MKP-1expression and further activated ERK1/2 pathway to suppress the progress of oxidative stress, inflammation and necrosis, contributing to the survival of skin flap injured by I/R. Thus, D-allose was promising in the transplantation of skin flap.

## Background

Flap transplantation technology is an important technical mean in the field of orthopedics (Picard-Ami et al. 1991; Tu et al. 2019; Varghese et al. 2019). It is widely used in other special fields such as orthopedics, microsurgery, oral and maxillofacial surgery, ophthalmology, etc. (Picard-Ami et al. 1991; Tu et al. 2019; Varghese et al. 2019). However, flap transplanted often sustains ischemia and hypoxia for a period of time, and then reperfusion of blood after ischemia, leading to an inevitably damage to the flap tissue and even its survival (Picard-Ami et al. 1991; Tu et al. 2019; Varghese et al. 2019; Liu et al. 2019). Nowadays, it is believed that the production of oxygen free radicals, calcium overload, energy metabolism disorders, vascular endothelial cell dysfunction, cytokine or growth factor production, necrosis are the critical mechanisms in the I/R injury to skin flap (Chen et al. 2018; Zhang et al. 2016). At present, methods for improving flap survival include improving surgical technique, shortening operation time, drug treatment, etc. (Liu et al. 2019)., However, these methods are not available under all circumstances due to their uncontrollability and differences of individuals (Liu et al. 2019). Therefore, drugs dedicating to intervene the mechanism of I/R described above is an effective treatment approach for improving the survival rate of flaps. The traditional drug administration route makes the drug evenly distributed in the systemic circulatory system, the approach which lacks specificity to the flap tissue (Liu et al. 2019). Thus, before the drug reaches the flap, it must undergo the progresses such as the binding of plasma protein, liver catabolism, etc., and finally reach the skin to exert its pharmacological function (Liu et al. 2019). In order to improve the efficacy of drugs and reduce its systemic side-effects, it is necessary to research and develop novel drugs with a higher specificity.

D-allose is a typical rare sugar extracted from D-ribose, attracting the attention of researchers recently (Gao et al. 2011). Although D-allose is particularly rare in nature, in addition to its general characteristics of rare sugars, it also has important physiological activities such as lowering blood lipid and blood sugar concentration, reducing free radicals and improving intestinal flora (Gao et al. 2011). Studies have found that D-allose can induce programmed cell apoptosis in malignant tumor cells, reduce the production of oxygen free radicals in inflammatory reaction and inhibit leukocyte activation, exerting its anti-inflammatory effects to protect liver, kidney and retina from I/R injury (Mizote et al. 2011; Indo et al. 2014; Liu et al. 2014).

Interestingly, ERK signal pathway was noticed to be associated with the development of I/R injury (Choi et al. 2017; Jin et al. 2017). Given these information, we intended to investigate the function of D-allose in skin flap of rat followed by the injury of I/R and whether ERK signal pathway was involved in. Our study highly demonstrated that D-allose was promising in the prevention of I/R in the transplantation of skin flap.

## Methods

### Animal ethics

Following the guidelines of the China Council on Animal Care and Use, all experiments in the present study were performed and permitted by the ethic boarder of Ruihua Affiliated Hospital of Soochow University. Any inhumane behavior is not applied to the animals unless necessary.

### Establishment of I/R model for skin flap

Forty healthy Wistar rats that were 12–14 weeks, male, 250-350 g were obtained from the Model Animal Research Center of Nanjing University. The breeding and model establishment were conducted in no specific pathogen (SPF) grade laboratory of Ruihua Affiliated Hospital of Soochow University under the temperature of 18–25 °C and 40–70% humidity. Animals were lived in the day-night cycle of 12 to 12 h with the supplement of regular feed and fertilized water.

Rats were weighed and injected intraperitoneally with 40 mg/kg sodium pentobarbital solution. After the detecting of eyelash reflex, rats were placed on a bench with constant temperature and fixed. Following the removal of the hair on the back and disinfection, the back flap was picked up along the line designed with the vascular bundle of the lateral chest wall in order to make the flap was only connected to the body by the vascular bundle. After the back wound was covered with silica gel, the vascular bundle of the rats in IR group was clamped with a venous clamp while the rats in the control group were not clamped. Then, the flap was sutured in situ. During the clamping of blood supply of the flap, 2% of pentobarbital sodium at 20 mg/kg was administered every 3 h. After 6 h, the suture and clamp were removed. Flap was sutured in situ after the confirming of recovery of blood flow under the microscope. Based on the treatment of I/R group, rats in D-allose group were intraperitoneally injected with D-allose (0.4 mg/g) half an hour before surgery (Sato et al. 2009). Similarly, rats in the PD group was intraperitoneally injected with D-allose (0.4 mg/g) + ERK inhibitor PD-98059 (300 μg/kg) half an hour before surgery. On the 7th day after surgery, the flaps were photographed by a digital camera. The image-analysis system of Image-Pro Plus.V6.0 was used to measure the necrotic area of ​​the flaps and calculate the survival rate of flaps followed by the formula below: flap survival rate = flap survival area (cm^2^) / total flap area (cm^2^) × 100%. Rats in all groups were sacrificed by excessive anesthesia after the analysis for the next experiments.

### HE staining

Following the modeling of rat in groups aforementioned, skin flap was obtained and fixed immediately by 4% formaldehyde from rats after the perfusion of PBS via circulatory system. Next, skin flap were cut into piece in order to embed into paraffin after 24 h of fixing. Briefly, the pieces of flap tissue were dewaxed by xylene substitute (A5597, Sigma-Aldrich, Missouri, USA) and then stained with hematoxylin (#14166, CST, MA, USA) and eosin (E4009, Sigma-Aldrich, Missouri, USA), following the procedures of HE staining provided by manufacturer of solutions purchased. The structure and cells in the flap tissue were further observed and recorded.

### Elisa

Nitric oxide (NO), myeloperoxidase (MPO), malondialdehyde (MDA) and superoxide dismutase (SOD) levels in skin flap were determined by ELISA kits. Due to the fact that NO production can be estimated via quantizing the levels of NO_2_ and NO_3_, Nitric Oxide assay kit (Nitrate reductase method, A012–1-2, Nanjing Jiancheng Bioengineering Institute) was used to evaluate the levels of NO produced during I/R in skin flap. Similarly, MPO was measured by Myeloperoxidase assay kit (A044–1-1, Nanjing Jiancheng Bioengineering Institute), MDA by Malondialdehyde assay kit (TBA method, A003–1-2, Nanjing Jiancheng Bioengineering Institute) and SOD by Superoxide Dismutase assay kit (WST-1 method, A001–3-2, Nanjing Jiancheng Bioengineering Institute), following the protocol provided by manufacturer.

### qPCR

The total RNA in the skin flap from rat was collected right after the treatment of TRIzol reagent (15,596,018, Thermo Fisher, Waltham, USA). Next, cDNAs of MCP-1, TNF-α, IL-1β and IL-6 were synthesized by PrimeScript RT reagent kit (Takara Biotechnology Co., Ltd.,Dalian, China). The preparation of reaction system of qPCR was conducted following the formula below with FastStart Universal SYBR Green Master (Rox) kit (4,913,850,001, Roche, Shanghai, China): 5 μL cDNA template, 10 μL 2 × SYBER Green master mix, 1 μL forward primer (10 μM), reverse primer 1 μL (10 μM), 3 μL ddH2O. qPCR procedure was performed further in Bio-Rad IQ5 thermocycler (Bio-Rad, CA, USA) following the procedures below: 90 s at 95 °C; 30 s at 95 °C; 30 s at 65 °C; 40 s at 72 °C; 40 cycles for total. Relative mRNA levels were calculated by 2^-ΔΔ*Ct*^ method. The primers used in qPCR were listed in Table 1.

**Table 1:** primers used in the study

Primer name	Sequence (5′-3′)
MCP-1 Forward	CGCTCAGCCAGATGCAATCAAT
MCP-1 Backward	CTTCTTTGGGACACTTGCTGC
TNF-α Forward	CCCATGTTGTAGCAAACCCTC
TNF-α Backward	TATCTCTCAGCTCCACGCCA
IL-1β Forward	CCACCTCCAGGGACAGGATA
IL-1β Backward	TGGGATCTACACTCTCCAGC
IL-6 Forward	CAATGAGGAGACTTGCCTGG
IL-6 Backward	TGGGTCAGGGGTGGTTATTG
GAPDH Forward	CGCTTCACGAATTTGCGTGTCAT
GAPDH Backward	GAAGATGGTGATGGGATTTC

### Western blot

Total protein in skin flap was collected by RIPA lysate (R0278, Sigma-Aldrich, Missouri, USA) mixed with protease inhibitor (S8830, Sigma-Aldrich, Missouri, USA) in order to determine the protein levels of RIP1, RIP3, MLKL, PGAM5, Drp1, MKP-1, p-ERK1, p-ERK2, ERK1 and ERK2. The concentration of total protein was evaluated by BCA kit (Sigma-Aldrich, Missouri, USA) in the first place to estimate the loading volume of samples onto the SDS-PAGE for separation. Parameter for electrophoresis was 100 V for 2 h. PVDF membrane was used for the transferring of protein separated. Following the blocking of membrane for 60 min, primary antibodies against RIP1 (CST, #3493, 78 kD, 1:1000), RIP3 (Abcam, ab56164, 57 kD, 1:1000), MLKL (CST, #37705, 54 kD, 1:1000), PGAM5 (Abcam, ab126534, 32 kD, 1:1000), Drp1 (Abcam, ab56788, 82 kD, 1:1000), MKP-1 (CST, #3493,78 kD, 1:1000), p-ERK1/2 (Abcam, ab50011,42–44 kD, 1:1000), ERK1/2 (Abcam, ab17942, 42–44 kD), GAPDH (Abcam, ab8245, 36kD, 1:2000) were incubated on the membrane at 4 °C overnight. Next day, after the washing of membrane for 3 times, primary antibodies were probed by Goat anti-rabbit secondary antibody (Abcam, ab205718, 1:2000) and developed by ECL (#6883, SignalFire™ ECL Reagent) after washing by PBST (PBS with 0.1% Tween).

### Statistics

Data collected from the present study was gathered into Graphpad prism 8.0 software. Comparisons within 4 groups were conducted by One-way analysis of variance. Tukey’s test was for comparison between any two means. P less than 0.05 was set as statistical significant.

## Results

### D-allose raised the survival rate of skin flap injured by I/R

**Fig. 1:**
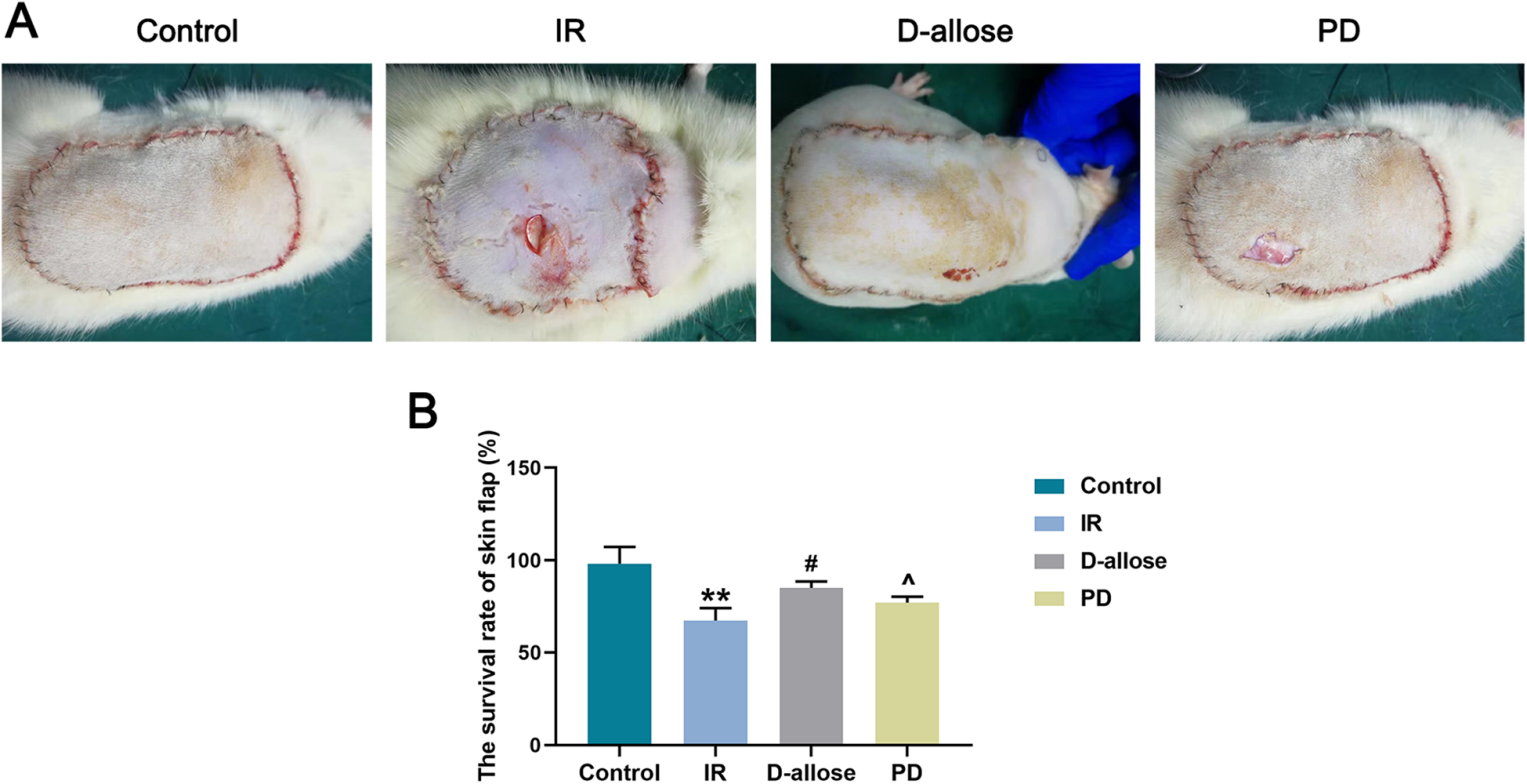
D-allose promoted the recovery and raised the survival rate of skin flap injured by I/R. **a** Pathological conditions of skin flap in Control, IR, D-allose and PD groups; **b** The survival rate of skin flap (%) in each group. Bars indicated means ± standard deviation (SD). ^**^P < 0.01 vs. Control; ^#^*P* < 0.05 vs. IR; ^^^*P* < 0.05 vs. D-allose

To observe whether D-allose was benefit for the survival of skin flap following the injury of I/R, the morphologic alteration of skin as well as the survival rate of skin flap was determined. Generally, in Control group, the edges of the suture were slightly red and swollen. In detail, on the third day, the redness on the skin was gradually subsided. On the fifth day, there was no swelling at the suture. On the 7th day, the flaps were almost all survived. The flaps were soft with the hair grows well on the surface. On the contrary, in IR group, the flaps were pale and marginal oozing was less. After 4–5 h of ischemia, the flap turned blue-purple with swollen and exudate. After reperfusion, the flap began to turn to dark with an aggravated swelling on the surface after 24–48 h. On the 3rd day, the swelling gradually subsided but inflammatory exudation could be seen at the suture. On the 5th day, the necrosis area of the flap was becoming larger, and some of the flaps were leather-like or hardened. No obvious hair growth was found as well. The early ischemic manifestations in D-allose group were similar to those of IR group. However, on the 3rd day, the swelling subsided obviously, and the color of the flap turned to normal. On the 5th day, there’s no obvious swelling in the flap but with a little inflammation on the suture edge. On the 7th day, most of the flap survived, and a small extent of necrosis was shown on the suture edge. The growth of hair was good on the surface of skin. Similar outcomes were demonstrated in PD group with a larger necrosis area in the flap compared to that in D-allose group (Fig. 1a). In addition, the survival rate of skin flap (%) in IR group was less than that in Control group while it was the highest in D-allose group compared to that in IR or PD group (Fig. 1b, ^**^P < 0.01; ^#^*P*, ^^^*P* < 0.05). Taken together, D-allose might be benefit for the surviving of flap after the injury of I/R, the trend which could reversed partially due to the treatment of ERK1/2 inhibitor.

### D-allose protected skin flap from the injury of I/R

**Fig. 2:**
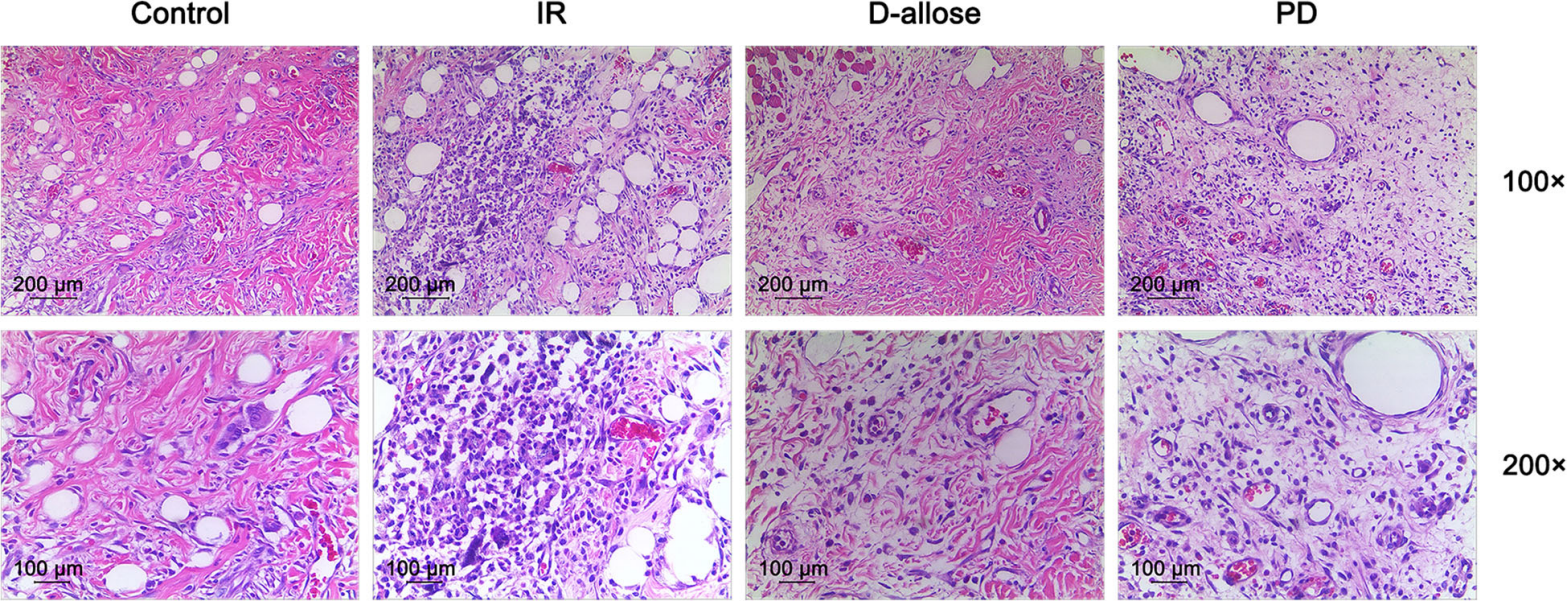
D-allose alleviated pathological condition of skin flap injured by I/R in Control, IR, D-allose and PD groups

Further, we observed the histopathological alteration in the skip flap following the injury of I/R. In control group, the stratum corneum and subcutaneous tissue structure of the flap were normal without obvious edema. A small amount of neutrophils are scattered in the interstitial space, and the fiber was neatly arranged with an intact wall of the blood vessel. However, in IR group, there were exfoliation and edema in part of the epidermis. The structure of fiber was fractured and arranged disorderly. Infiltrations of erythrocyte and inflammatory cell were also could be seen in the flap. Reversely, in D-allose group, the overall structure of the flap tissue is complete with a mild edema and the infiltration of inflammatory cells. The fiber structure was basically neat with the existence of the integrity of vessel wall. The infiltration of erythrocyte was barely seen in the flap as well. Similar performance was seen in the skin flap from PD group with a little more infiltrated inflammatory cells compared to that in D-allose group (Fig. 2). Though without Ly6G or Gr-1 histology, D-allose was shown being able to alleviate the injury of skin flap injured by I/R. However, the treatment of ERK1/2 inhibitor could counteract such a trend to some extent.

### D-allose suppressed oxidative stress in skin flap injured by I/R

**Fig. 3:**
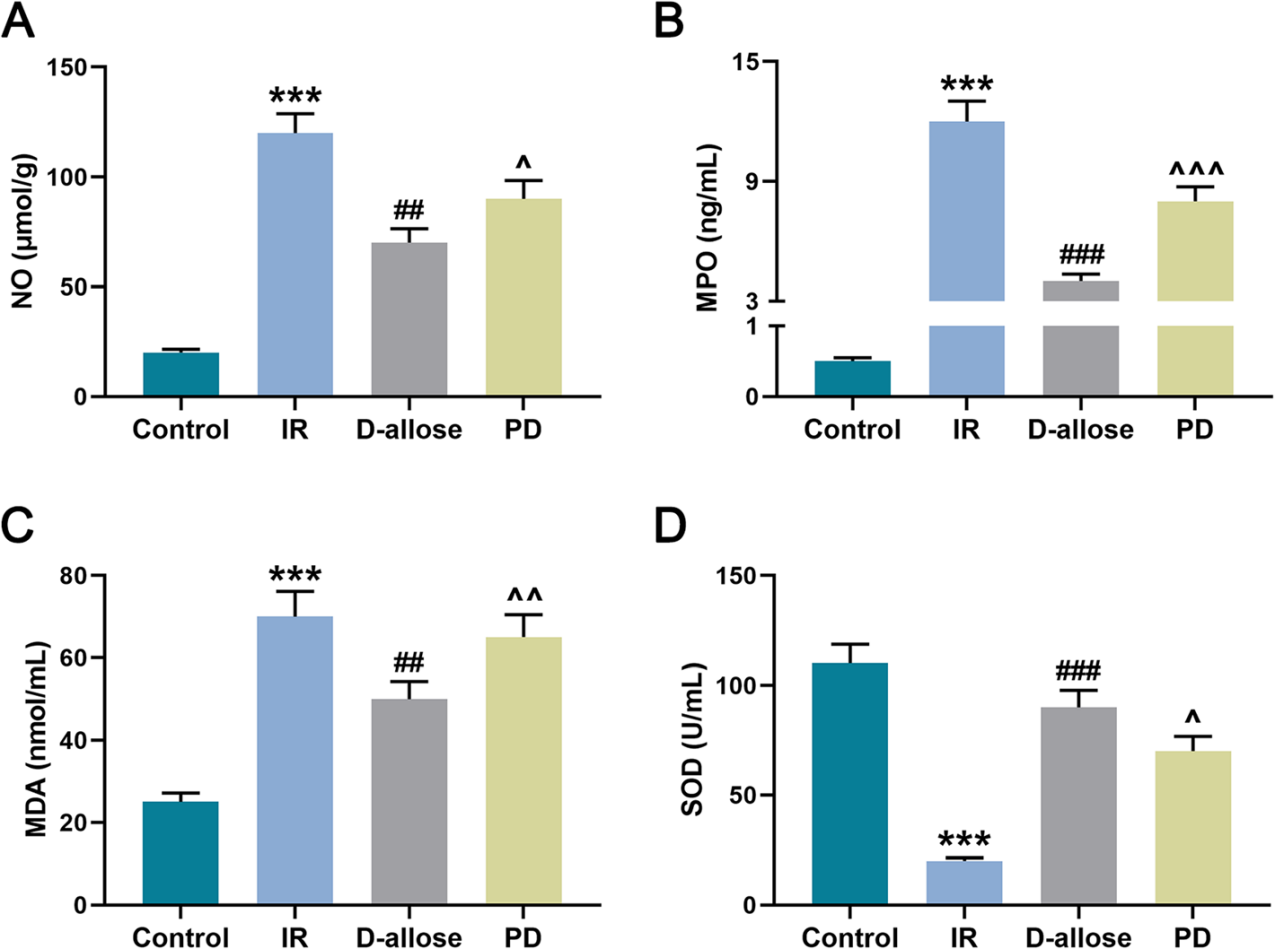
D-allose suppressed oxidative stress in skin flap injured by I/R. **a** NO (μmol/g) level in skin flap was measured by ELISA in Control, IR, D-allose and PD groups; **b** MPO (ng/mL) level in skin flap was measured by ELISA in each group; **c** MDA (nmol/mL) level in skin flap was measured by ELISA in each group; **d** SOD (U/mL) level in skin flap was measured by ELISA in each group. Bars indicated means ±SD. ^***^P < 0.001 vs. Control; ^###^*P* < 0.001, ^##^*P* < 0.01 vs. IR; ^^^^^*P* < 0.001, ^^^^*P* < 0.01, ^^^*P* < 0.05 vs. D-allose

Moreover, the status of oxidative stress was determined as well right after the injury of I/R given to the skin flap of rat. Here, we discovered that the NO, MPO and MDA levels in the skin flap in IR group was significantly higher than that in Control group while they were the lowest compared to that in IR as well as in PD groups (Fig. 3a-c, ^***^P, ^###^*P*, ^^^^^*P* < 0.001; ^##^*P*, ^^^^*P* < 0.01; ^^^*P* < 0.05). However, the SOD level in IR group was dramatically decreased compared to that in Control group while it was the highest in D-allose group compared to that in IR and PD groups (Fig. 3d, ^***^P, ^###^*P* < 0.001; ^^^*P* < 0.05). It suggested that the oxidative stress triggered by I/R could be suppressed by D-allose in skin flap, and ERK1/2 signal pathway might be involved.

### D-allose decreased inflammatory factors in skin flap injured by I/R

**Fig. 4:**
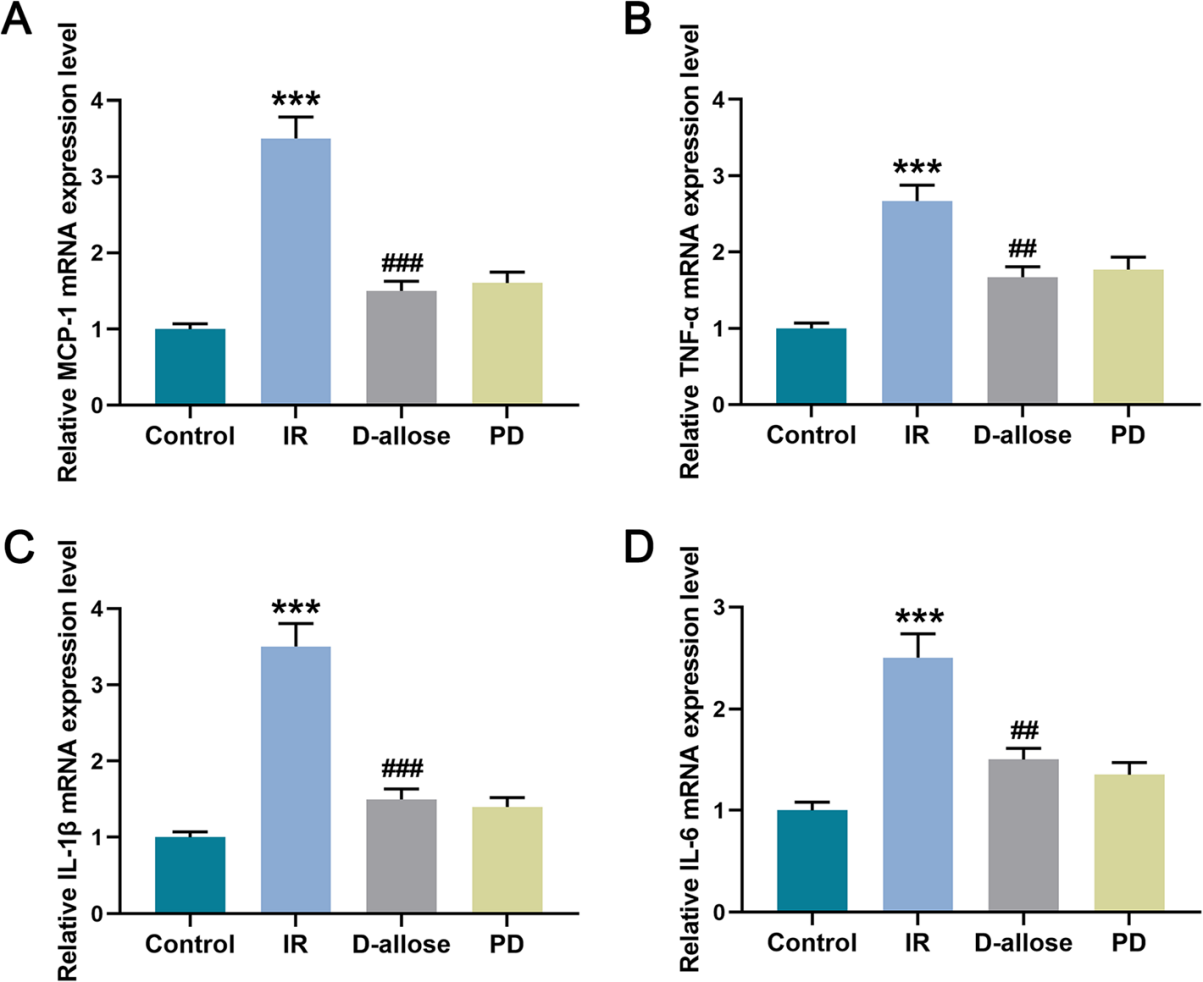
D-allose decreased inflammatory factors in skin flap injured by I/R. **a** Relative MCP-1 mRNA expression level in skin flap was measured by RT-qPCR in Control, IR, D-allose and PD groups; **b** Relative TNF-α mRNA expression level in skin flap was measured by RT-qPCR in each group; **c** Relative IL-1β mRNA expression level in skin flap was measured by RT-qPCR in each group; **d** Relative IL-6 mRNA expression level in skin flap was measured by RT-qPCR in each group. Bars indicated means ±SD. ^***^P < 0.001 vs. Control; ^###^*P* < 0.001, ^##^*P* < 0.01 vs. IR

Simultaneously, the alterations of the levels of inflammatory factors in skin flap were also evaluated. We found that MCP-1, TNF-α, IL-1β and IL-6 levels were elevated in IR group compared to that in Control group while they were decreased in D-allose group when they compared to that in IR group. However, there’s no significant difference D-allose and PD group on these levels (Fig. 4a-d, ^***^P, ^###^*P* < 0.001; ^##^*P* < 0.01). Thus, what could be speculated was that D-allose could down-regulate the raised inflammatory factors such as MCP-1, TNF-α, IL-1β and IL-6. However, ERK1/2 signal pathway might not participated in the mechanism of D-allose in the modulation of the releasing of inflammatory factors in I/R injury.

### D-allose inhibited the deactivation of ERK1/2 signal pathway and the expressions of genes related to programmed necrosis in skin flap injured by I/R

**Fig. 5:**
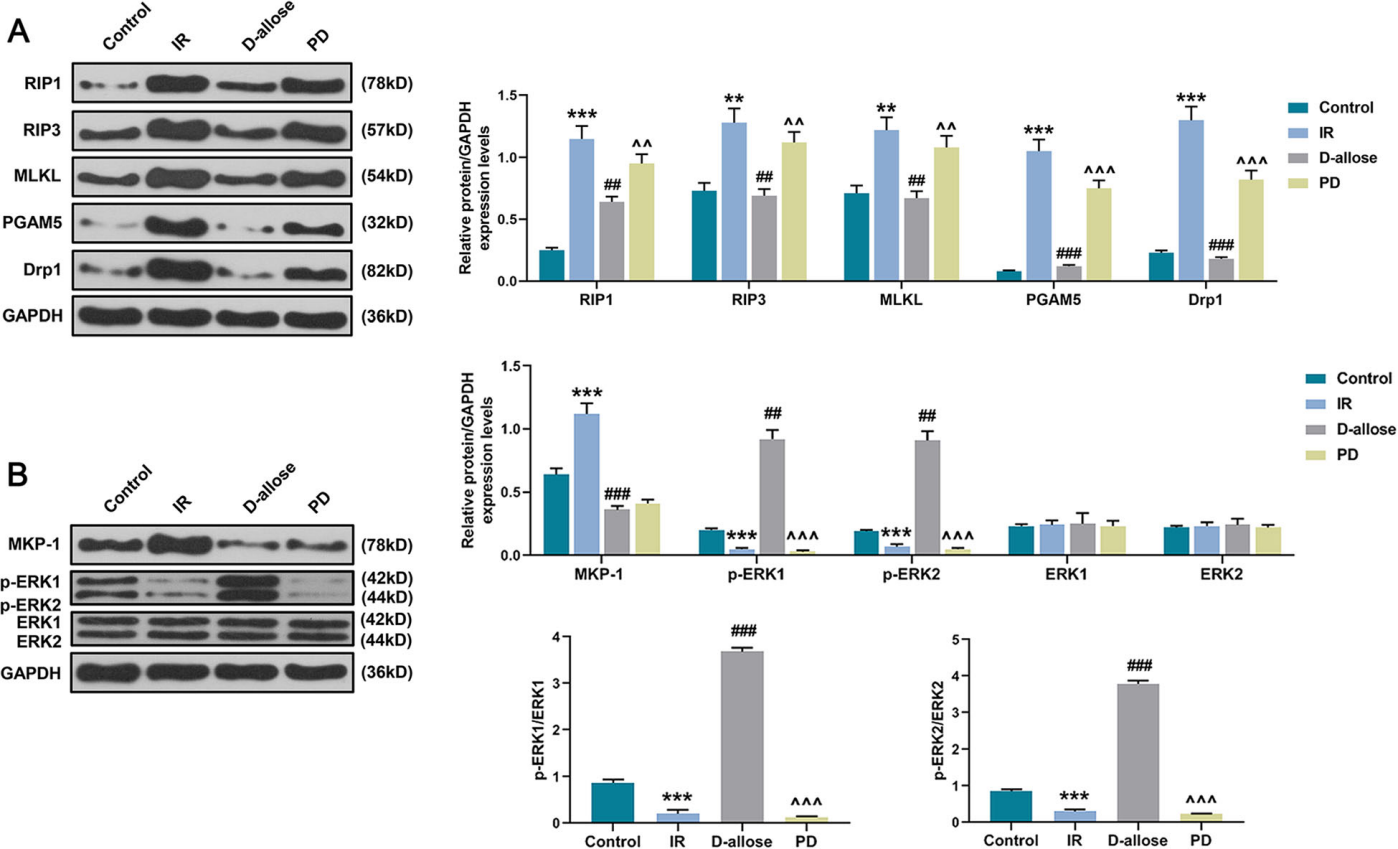
D-allose inhibited the deactivation of ERK1/2 signal pathway and the expressions of genes related to programmed necrosis in skin flap injured by I/R. **a** Protein blots and relative protein expressions of RIP1, RIP3, MLKL, PGAM5 and Drp1 were determined by Western blot in Control, IR, D-allose and PD groups; **b** Protein blots and relative protein expressions of MKP-1, p-ERK1/2 and ERK1/2 were determined by Western blot in each group. Bars indicated means ±SD. ^***^P < 0.001, ^**^P < 0.01 vs. Control; ^###^*P* < 0.001, ^##^*P* < 0.01 vs. IR; ^^^^^*P* < 0.001, ^^^^*P* < 0.01 vs. D-allose

In depth, we estimated the expressions of genes related to programmed necrosis as well as ERK1/2 signal pathway following the treatment of D-allose and ERK1/2 inhibitor in skin flap injured by I/R. Here, we found that the protein expressions of RIP1, RIP3, MLKL, PGAM5 and Drp1 were up-regulated in IR group compared to that in Control while they were the lowest in D-allose group compared to that in IR and PD groups (Fig. 5a, ^***^P, ^###^P, ^^^^^*P* < 0.001; ^**^P, ^##^P, ^^^^*P* < 0.01). Moreover, the protein expressions of MKP-1 was elevated while p-ERK1, p-ERK2, p-ERK1/ERK1 as well as p-ERK2/ERK2 were decreased in IR group (Fig. 5b, ^***^P < 0.001). In addition, when the expressions of p-ERK1, p-ERK2, p-ERK1/ERK1 as well as p-ERK2/ERK2 were the highest in D-allose group compared to that in IR and PD groups, MKP-1 expression in D-allose group was only significantly decreased compared to that in IR group but not to that in PD group (Fig. 5b, ^###^P, ^^^^^P < 0.001; ^##^*P* < 0.01). Given that, D-allose contributed to the decreasing of expressions of programmed necrosis genes as well as the activation of ERK1/2 signal pathway.

## Discussion

Previous study showed that the rare sugar D-allose had a reducing effect against ischemia-reperfusion injury on the rat abdominal skin island flap model (Muneuchi et al. 2013). In our study, the mechanism of D-allose’s action was identified and cutaneous skin flap model was applied. D-allose was proved to be able to alleviate the pathological state of skin flap injured by I/R as well as to suppress the oxidative stress, inflammatory response and programmed necrosis with the increasing of the survival rate of skin flap and the activation of ERK1/2 signal pathway. The results revealed demonstrated that D-allose might be an effective drug in the prevention of I/R injury toward skin flap in the practice of the treatment of thermal injury or plastic surgery. It might be a limitation not showing the results of vehicle control against D-allose or PD, like some articles (Sato et al. 2009; Gao et al. 2013).

Interestingly, we revealed that D-allose was effective in the prevention of skin flap from I/R, resulting in a better condition of pathological status as well as the elevation of the survival rate of skin flap. Leng, X. et al. demonstrated the performance of pathological changes in skin flap model of I/R in rat, the outcome which was in line with the results in the present study, indicating the success of the establishment of model. The pathological characteristics presented by Dong, X. H. et al. (Dong et al. 2019). were consistent with our results as well. However, D-allose alleviated such an injury by I/R, arousing the interests of us to further investigate the mechanism of D-allose.

NO is a vascular endothelial relaxing factor, which is a highly reactive free radical in the body (Zhang et al. 2019). It is also an important messenger and effector molecule with a wide range of physiological effects in the body (Zhang et al. 2019). MPO is considered to be a specific marker for neutrophils (Khan et al. 2018). During the progression of I/R pathology, neutrophils are activated and MPO is released into phagosomes to promote the secretion of reactive oxygen species (Khan et al. 2018). MDA and SOD are commonly used indicators of oxidative stress (Liu et al. 2015; Gorski et al. 2003; Du et al. 2017). MDA is the final product of oxidative reaction between ROS and biofilm lipid components, and is positively correlated with oxidative stress and lipid peroxidation (Liu et al. 2015; Gorski et al. 2003; Du et al. 2017). It can directly reflect the degree of lipid peroxidation in the body cells and indirectly reflect the severity of the cells being attacked by free radicals (Liu et al. 2015; Gorski et al. 2003; Du et al. 2017). SOD is the main redox regulating enzyme of tissue cells, which can catalyze the scavenging reaction of superoxide free radicals in living organisms (Liu et al. 2015; Gorski et al. 2003; Du et al. 2017). In our study, the levels of NO, MPO and MDA were heavily raised with the significant decrease of SOD. However, the usage of D-allose could reverse such a trend to some extent that the levels of NO, MOP and MDA were down-regulated with D-allose treatment. Nakamura, T. et al. noted that there was an anti-oxidative effects of D-allose which could further induce neuroprotection in focal cerebral ischemia (Nakamura et al. 2011). Coincidentally, Hirooka, K. et al. also mentioned that during the insult of ischemia, D-allose could attenuate oxidative stress in neurons (Hirooka et al. 2006). Thus, the mediation of oxidative stress might be involved in the mechanism of D-allose in the treatment of I/R in skin flap. It might be a limitation not applying TUNEL assay showing the apoptotic cells contents, which might be studied in the future.

MCP-1 acts as a monocyte chemotactic protein, mainly secreted by monocytes, macrophages and dendritic cells (Anders et al. 2018; Du et al. 2016). When an inflammatory reaction occurs, it activates the chemotaxis of monocytes/macrophages, activates the expression of leukocyte adhesion molecule-1, induces the production of the cytokine IL-1, IL-6, and regulates expression of specific adhesion molecules such as CDllc and CDllb on the surface of monocyte (Anders et al. 2018; Du et al. 2016). Besides, as an important inflammatory initiating factor, TNF-α has the cytotoxic and multi-proinflammatory effects, indicating the activation of inflammatory (Zhang et al. 2011). Here, we discovered that the levels of MCP-1, TNF-α, IL-1β and IL-6 were increased followed by the treatment of I/R in skin flap. However, D-allose could offset such a trend by I/R, decreasing the levels of inflammatory factors we measured. Shinohara, N. et al. mentioned that the overexpression of inflammatory cytokines could be depressed by D-allose in Gerbil with cerebral ischemia/reperfusion injury (Shinohara et al. 2016). Moreover, Huang, T. et al. noticed that the blood brain barrier could be protected by D-allose via mediating anti-inflammatory pathway when I/R injury occurred in mice (Huang et al. 1642). Given that, D-allose was proved to be negative to the releasing of inflammatory factors.

Proverbially, the RIP1/RIP3/MLKL pathway mediates the programmed necrosis of cells (Cho et al. 2009; He et al. 2009; Wang et al. 2014; Zhang et al. 2009). RIP1 and RIP3 are both necessary factors for necrosis, and the importance of RIP3 is more specific and critical (Cho et al. 2009; He et al. 2009; Wang et al. 2014; Zhang et al. 2009). RIP3 is a key protein molecule for the conversion of two different cell death pathways induced by TNF-α receptor (Cho et al. 2009; He et al. 2009; Wang et al. 2014; Zhang et al. 2009). Through the activation of RIP3, it can directly interact with the downstream three energy metabolism-related proteases glycogen phosphorylase (PYGL), glutamate-ammonia ligase (GLUL) and glutamate dehydrogenase 1 (GLUD1), resulting in the producing of a large number of oxygen free radicals, leading to the necrosis of cells (Cho et al. 2009; He et al. 2009; Wang et al. 2014; Zhang et al. 2009). MLKL is a key signaling molecule in the downstream of RIP3 and TNF-α receptor-mediated programmed cell necrosis pathway (Cho et al. 2009; He et al. 2009; Wang et al. 2014; Zhang et al. 2009). Phosphorylated MLKL binds to PGAM5 phosphatase on the surface of mitochondria and to Drp1 in the cytosol (Lin et al. 2013; Moriwaki et al. 2016). PGAM5 dephosphorylates Drp1 to promote its activation and ultimately leads to the fragmentation of mitochondria and necrotic apoptosis of cells (Lin et al. 2013; Moriwaki et al. 2016). Here, we found that I/R injury in skin flap could result in the increasing of RIP1, RIP3, MLKL, PGAM5 and Drp1 levels, suggesting that the necrosis of cells was promoted. However, D-allose counteracted such a trend, resisting the activation of programmed necrosis of cells in skin flap. Report by Hossain, M. A. et al. revealed that D-allose decreased the degree of necrosis in rat liver injured by I/R (Hossain et al. 2003). Taken together, D-allose was benefit for the resistance of necrosis triggered by I/R in skin flap.

Furthermore, MKP-1 level was elevated companied by the increasing of p-ERK1, p-ERK2, ERK1, ERK2, p-ERK1/ERK1 and p-ERK2/ERK2. MKP-1 is a specific negative regulator of the ERK pathway that blocks substrate activity and promotes serine/threonine and tyrosine phosphorylation (Nithianandarajah-Jones et al. 2012). It has been reported that the imbalance between MKP-1 and ERK may be an important cause of uncontrolled proliferation and apoptosis of cells (Nithianandarajah-Jones et al. 2012). Thus, during the status of I/R, ERK1/2 pathway was deactivated whereas D-allose could counteract such a trend, inhibiting the deactivating of ERK1/2 pathway. In addition, PD-98059, the inhibitor of ERK1/2, could reverse the effect of D-allose on the decreasing of the survival rate of skin flap as well as the increasing of the oxidative stress and necrosis expect the levels of inflammatory factors. Choi, D. E. et al. revealed that ERK activation was critical against renal I/R injury (Choi et al. 2017). Jin, Q. et al. also noticed that estradiol mediated ERK pathway via inhibiting MKP-1 expression, reducing the injury of axial flap by I/R (Jin et al. 2017). Given these evidences, ERK pathway related molecules, especially MKP-1, might be involved in the mechanism of D-allose in the protective effect of I/R in the skin flap. In addition, as MKP-1 also regulates P38 and JNK pathway, whether P38 and JNK pathway was involved in the mechanism of D-allose relating to skin flap I/R would be further studied in the future.

## Conclusions

In summary, D-allose might exert its protective function via inhibiting MKP-1expression and further suppress the progress of oxidative stress, inflammation and necrosis, contributing to the survival of skin flap injured by I/R. Based on this finding, D-allose is promising in the practice of transplantation of skin flap, protecting the damage of I/R when necessary.

## Data Availability

The analysed data sets generated during the study are available from the corresponding author on reasonable request.

## Abbreviations

I/R: Ischemia/reperfusion
MDA: Malondialdehyde
MPO: Myeloperoxidase
NO: Nitric oxide
SOD: Superoxide dismutase
SPF: Specific pathogen
